# Butyrate-associated antiproliferative and antitumor effects of *Eubacterium limosum* KGMB01548 in colorectal cancer

**DOI:** 10.3389/fmicb.2026.1973432

**Published:** 2026-10-06

**Authors:** Seung Yeob Yu, Jeong Eun Bak, Byeong Seob Oh, Seoung Woo Ryu, Hyebin Park, Dong Jin Yu, Jiyoung Lee, Ji-Sun Kim, Ju Huck Lee

**Affiliations:** 1 Korean Collection for Type Cultures, Biological Resource Center, Korea Research Institute of Bioscience and Biotechnology, Jeongeup, Republic of Korea; 2 University of Science and Technology (UST), Daejeon, Republic of Korea; 3 Department of Biomedical Science, Sunchon National University, Suncheon, Republic of Korea

**Keywords:** apoptosis, butyrate, cell-free supernatant, colorectal cancer, *Eubacterium limosum*, gut microbiota, histone acetylation

## Abstract

**Introduction:**

Recent work has shown that *Eubacterium limosum* suppresses colorectal cancer (CRC), but the contribution and cellular actions of its short-chain fatty acids remain incompletely defined.

**Methods:**

We characterized *E. limosum* KGMB01548, a human gut isolate, and examined the antitumor activity of its cell-free culture supernatant (ElCFS).

**Results:**

ElCFS preferentially inhibited the proliferation of human HCT116 and murine CT26 CRC cells while having little effect on non-malignant CCD 841 CoN colonic cells. The activity persisted after heat and enzymatic treatments, and pH-dependent liquid–liquid extraction indicated that a small organic acid was a major bioactive component. High-performance liquid chromatography detected 13.77 mM butyrate and 23.33 mM acetate in ElCFS. At approximately the concentration delivered by 10% ElCFS, sodium butyrate (NaB; 1.3 mM) produced comparable antiproliferative activity, whereas sodium acetate did not, supporting butyrate as a major candidate contributor to ElCFS activity. ElCFS and approximately concentration-matched NaB increased histone H3 acetylation, consistent with histone deacetylase inhibition, and induced apoptosis, as evidenced by PARP and caspase-3 cleavage and p53 phosphorylation. Their antiproliferative effects were markedly attenuated under glucose-depleted conditions. In a CT26 syngeneic tumor model, peritumoral administration of ElCFS reduced tumor growth, indicating that secreted *E. limosum* metabolites retained local antitumor activity outside the intestinal route. Oral pre-administration of live *E. limosum* also reduced subsequent tumor burden. Furthermore, 16S rRNA gene profiling suggested modest changes in several putative short-chain-fatty-acid-producing taxa and in PICRUSt2-predicted butyrate-biosynthesis potential, without a major change in alpha diversity.

**Discussion:**

Together, these findings link *E. limosum* KGMB01548 to butyrate-associated histone acetylation, apoptosis, glucose-sensitive antiproliferative activity, and tumor suppression, supporting further investigation of this gut commensal and its secreted metabolites as microbiome-derived approaches for CRC control.

## 1 Introduction

Colorectal cancer (CRC) is one of the most commonly diagnosed malignancies and remains a leading cause of cancer-related mortality (Siegel et al., 2023). Although inherited susceptibility contributes to a subset of CRC cases, most cases are sporadic and are influenced by non-genetic factors, including diet, lifestyle, inflammation, and other environmental exposures (Rebersek, 2021). Among these factors, the gut microbiota has received increasing attention because its composition and functional activity differ between patients with CRC and healthy individuals (Clavel et al., 2022). Several bacterial taxa, including *Parvimonas micra* and *Fusobacterium nucleatum*, are repeatedly enriched in CRC-associated microbial communities and have been investigated as microbial biomarkers and potential contributors to tumor development (Zhao et al., 2021; Zepeda-Rivera et al., 2024).

The gut microbiota contributes to host nutrient metabolism, epithelial barrier function, immune development, and colonization resistance (Cheng et al., 2020; Zheng et al., 2020; Olofsson and Bäckhed, 2022). Many of these effects are mediated by microbial metabolites, which provide a biochemical interface between microorganisms and host cells (Jansma and El Aidy, 2021). When this ecosystem is disrupted, dysbiosis can alter the microbial metabolism of dietary carbohydrates, proteins, and lipids, thereby modifying the intestinal chemical environment in inflammatory bowel disease and CRC (Heinken et al., 2021; Li et al., 2022). Consistent with this concept, several studies have reported lower fecal concentrations of short-chain fatty acids (SCFAs), sometimes accompanied by increased fecal pH, in patients with CRC than in healthy controls (Sobhani et al., 2011; Ohigashi et al., 2013; Yusuf et al., 2019).

Acetate, propionate, and butyrate are major microbiota-derived SCFAs produced through the anaerobic fermentation of dietary fiber by commensal bacteria, including members of *Faecalibacterium*, *Roseburia*, *Ruminococcus*, and *Eubacterium* (Morrison and Preston, 2016; Nogal et al., 2021; Zou et al., 2021). Butyrate is preferentially utilized by colonocytes and contributes to epithelial barrier integrity, inflammatory regulation, and colonic homeostasis (Hodgkinson et al., 2023). In CRC cells, butyrate can inhibit histone deacetylases (HDACs), increase histone acetylation, and alter transcriptional programs controlling cell proliferation, cell-cycle progression, and apoptosis (Tan et al., 2014). Consistent with the potential importance of SCFA metabolism in CRC, several SCFA-producing taxa are depleted in CRC-associated gut microbial communities (Chen et al., 2012; Wang et al., 2012; Yusuf et al., 2019). Nevertheless, because bacterial culture supernatants contain complex mixtures of bioactive metabolites, the mere detection of butyrate does not establish whether, or to what extent, it accounts for the observed antiproliferative activity.

Members of the genus *Eubacterium* are phylogenetically diverse obligate anaerobes that contribute to multiple metabolic functions in the human gut (Wade, 2006). Several species, including *E. rectale* and *E. limosum*, can produce butyrate and have been associated with the maintenance of epithelial barrier function and the regulation of intestinal inflammation (Kanauchi et al., 2006; Canani et al., 2011; Akhtar et al., 2022). Recently, Lu et al. demonstrated that *E. limosum* El1405 suppressed CRC cell proliferation and tumor growth through a broad combination of mechanisms, including the production of several bioactive metabolites—such as indole-3-lactic acid, indole-3-acetic acid, L-arginine, and butyrate—together with alterations in the intestinal and intratumoral microbiota and the tumor microenvironment (Lu et al., 2025). These findings established a multi-metabolite and ecosystem-level framework for the anti-CRC activity of *E. limosum*. Nevertheless, because several bioactive metabolites coexist in the cell-free culture supernatant, the extent to which butyrate at the concentration present in the supernatant contributes to its antiproliferative activity remains unresolved. It is also unclear whether the supernatant recapitulates butyrate-associated histone acetylation under defined metabolic conditions and whether its secreted metabolites retain local tumor-suppressive activity when administered locally rather than through the gastrointestinal tract. Addressing these questions using an independent human-derived strain would provide complementary mechanistic and strain-level evidence.

In the present study, we investigated *E. limosum* KGMB01548, isolated from the feces of a healthy Korean donor, and characterized the antitumor activity of its cell-free culture supernatant (ElCFS). We first evaluated the preferential antiproliferative activity of ElCFS against human HCT116 and murine CT26 CRC cells relative to non-malignant CCD 841 CoN colonic cells. We then combined thermal and enzymatic stability assays, pH-dependent liquid–liquid extraction, high-performance liquid chromatography, and genome-based pathway analysis with approximately concentration-matched comparisons of sodium butyrate (NaB) and sodium acetate. To examine the associated host-cell response, we assessed histone H3 acetylation, apoptosis, cell-cycle distribution, and the dependence of antiproliferative activity on glucose availability. *In vivo*, ElCFS was administered peritumorally in a CT26 syngeneic tumor model to determine whether the secreted metabolites retained local tumor-suppressive activity without oral delivery. Oral pre-administration of live *E. limosum* and 16S rRNA gene profiling were additionally used to evaluate tumor suppression and associated changes in the gut microbial community. By integrating microbial, biochemical, cellular, and *in vivo* analyses, this study aimed to characterize butyrate-associated cellular and biochemical features of *E. limosum* activity and to provide a basis for further evaluation of this strain and its secreted metabolites as microbiome-derived approaches for CRC control.

## 2 Materials and methods

### 2.1 Cell culture

HCT116 human colorectal carcinoma cells were obtained from the Korean Collection for Type Cultures (KCTC; Jeongeup, Republic of Korea). CCD 841 CoN human colonic epithelial cells (ATCC CRL-1790) and CT26 murine colorectal carcinoma cells (ATCC CRL-2638) were obtained from the American Type Culture Collection (ATCC; VA, USA). All cell lines were maintained in Dulbecco’s modified Eagle’s medium (DMEM; Welgene, Gyeongsan, Republic of Korea) supplemented with 10% fetal bovine serum (FBS; Gibco, NY, USA) and 1% antibiotic–antimycotic solution (100×; Welgene). For glucose-depletion experiments, cells were cultured in glucose-free DMEM (Gibco) supplemented with 10% FBS and 1% antibiotic–antimycotic solution. All cells were maintained at 37 °C in a humidified incubator with 5% CO₂.

### 2.2 Bacterial culture and preparation of ElCFS

*Eubacterium limosum* KGMB01548 was isolated from a fecal sample obtained from a healthy Korean individual, as described previously (Oh et al., 2021). The taxonomic identity of the strain was confirmed by 16S rRNA gene sequencing and phylogenetic analysis, together with phenotypic characterization. All bacterial isolation and cultivation procedures were performed in an anaerobic chamber (Coy Laboratory Products, MI, USA) under an atmosphere of 86% N₂, 7% CO₂, and 7% H₂. The strain was routinely subcultured every 2 days on tryptic soy agar (BD, NJ, USA) supplemented with 5% sheep blood. Bacterial stocks were stored at −80 °C in sterile 10% (v/v) skim milk. The *E. limosum* cell-free supernatant (ElCFS) was prepared as described previously, with minor modifications (Oh et al., 2021). Briefly, *E. limosum* KGMB01548 was inoculated into 20 mL of reinforced clostridial medium (RCM; MBcell, Seoul, Republic of Korea) and cultured anaerobically at 37 °C for 48 h. The bacterial culture was centrifuged at 6,000 × *g* for 30 min, and the resulting supernatant was collected. The pH of the supernatant was adjusted to 6.8 using 1 M NaOH to minimize the effect of medium acidification on the subsequent assays. The pH-adjusted supernatant was then passed through a 0.22-μm hydrophilic polyethersulfone membrane syringe filter to remove residual bacterial cells. Uninoculated RCM was incubated and processed under the same conditions, including pH adjustment, centrifugation, and filtration, and was used as the matched medium control. ElCFS and the processed RCM control were stored at −20 °C until use.

### 2.3 Crystal violet staining and viable cell counting

HCT116 cells were seeded in 6-well plates at a density of 5 × 10^4^ cells per well and allowed to adhere for 24 h. The culture medium was then replaced with fresh complete DMEM containing either 10% (v/v) RCM or 10% (v/v) ElCFS. Cells were incubated for an additional 72 h at 37 °C in a humidified atmosphere containing 5% CO₂. For crystal violet staining, cells were washed twice with Dulbecco’s phosphate-buffered saline (DPBS), fixed with 4% paraformaldehyde, and stained with 1% crystal violet solution (Thermo Fisher Scientific, MA, USA). Excess stain was removed by washing, and the stained cells were imaged to visualize differences in cell density. For viable cell counting, HCT116 cells were seeded and treated under the same conditions. After 72 h, cells were harvested and stained with trypan blue solution (Sigma-Aldrich, Hesse, Germany). Viable cells were counted using a hemocytometer based on trypan blue exclusion. Experiments were performed independently three times.

### 2.4 MTT-based assessment of cell viability

The antiproliferative effects of ElCFS on HCT116, CT26, and CCD 841 CoN cells were evaluated using an MTT assay. Cells were seeded in 96-well plates at densities of 4 × 10^4^ HCT116 cells, 5 × 10^3^ CT26 cells, or 2 × 10^4^ CCD 841 CoN cells per well and allowed to adhere for 24 h. The culture medium was then replaced with fresh medium containing either 10% (v/v) RCM or 10% (v/v) ElCFS. For concentration-dependent experiments, cells were treated with the indicated concentrations of ElCFS. Cells were incubated for an additional 72 h at 37 °C in a humidified atmosphere containing 5% CO₂. After treatment, the medium was replaced with fresh DMEM containing 1% antibiotic–antimycotic solution and 10% (v/v) MTT stock solution prepared at 5 mg/mL, resulting in a final MTT concentration of 0.5 mg/mL. Following incubation for 2 h at 37 °C, the medium was removed, and the resulting formazan crystals were dissolved in 0.04 M HCl–isopropanol. Absorbance was measured at 595 nm using a Multiskan FC microplate reader (Thermo Fisher Scientific). Antiproliferative activity was calculated using the following equation:


$$\text{Anti}­\text{proliferative activity}\;(\%)=\frac{A_{\text{control}}-A_{\text{treatment}}}{A_{\text{control}}}\times 100$$

Where $A_{\text{treatment}}$ and $A_{\text{control}}$ represent the absorbance values of the treated group and its corresponding control group, respectively. To assess potential treatment-associated changes in culture-medium pH, assay media were prepared under the same conditions used for the cell experiments. The measured pH values were 7.98 for untreated DMEM, 7.68 for DMEM containing 10% (v/v) RCM, 7.68 for DMEM containing 10% (v/v) ElCFS, and 7.89 for DMEM containing 1.3 mM NaB. Both ElCFS and the matched RCM control were adjusted to pH 6.8 before addition to the culture medium. Sodium acetate and sodium butyrate, rather than the corresponding free acids, were used to minimize treatment-associated acidification. Osmolality of the treatment media was not directly measured.

### 2.5 Physicochemical characterization of ElCFS and quantification of SCFAs

To characterize the physicochemical properties of the antiproliferative components in ElCFS, heat stability, resistance to enzymatic treatment, solvent partitioning, and SCFA concentrations were evaluated. For the heat-stability assay, ElCFS and RCM were heated at 100 °C for 10 min and cooled to room temperature before cell treatment. To evaluate susceptibility to enzymatic degradation, ElCFS was treated with amylase, papain, trypsin, pepsin, chymotrypsin, proteinase K, or lipase (Sigma-Aldrich, Hesse, Germany) at 10 μL/mL for 1 h at 37 °C. Following enzymatic treatment, the samples were heated at 100 °C for 10 min to inactivate the enzymes and then cooled to room temperature before use. To examine the partitioning behavior of the antiproliferative components, ElCFS and RCM were subjected to liquid–liquid extraction using ethyl acetate (EtOAc). Briefly, 500 mL of each sample was mixed vigorously with an equal volume of EtOAc and incubated overnight at room temperature. The aqueous and organic phases were separated using a separatory funnel. The organic phase was evaporated to dryness using a rotary vacuum evaporator (EYELA, Tokyo, Japan) and reconstituted in 5 mL of EtOAc. The aqueous phase was evaporated under reduced pressure for 5 min to remove residual EtOAc. The extraction was performed at pH 6.8 and pH 2.5. Sample pH was adjusted before extraction using 1 M NaOH or 10 M HCl.

Acetate and butyrate concentrations in ElCFS and the aqueous fractions were determined by high-performance liquid chromatography (HPLC; Shimadzu, Kyoto, Japan). Samples were separated using an Aminex organic acid analysis column (Bio-Rad, CA, USA) with 5 mM H₂SO₄ as the isocratic mobile phase at a flow rate of 0.7 mL/min for 25 min. Eluted compounds were detected at 210 nm and identified by comparison of their retention times with those of authentic acetate and butyrate standards. Concentrations were calculated from calibration curves generated using known concentrations of the corresponding standards.

### 2.6 Flow cytometric analysis of apoptosis and cell-cycle distribution

Apoptosis was assessed using an Annexin V-FITC/PI apoptosis detection kit (Thermo Fisher Scientific) with minor modifications to a previously described method (Oh et al., 2021). HCT116 cells were seeded in 6-well plates at a density of 3 × 10^5^ cells per well and allowed to adhere for 24 h. The culture medium was then replaced with fresh medium containing 10% (v/v) RCM, 10% (v/v) ElCFS, or 1.3 mM NaB. Cells were incubated for an additional 72 h. Both floating and adherent cells were collected, with adherent cells detached using trypsin–EDTA. The collected cells were washed twice with cold DPBS and resuspended in Annexin V binding buffer. Cells were stained with Annexin V-FITC for 10–15 min at room temperature in the dark, followed by PI staining immediately before analysis. Apoptotic cell populations were analyzed using an Attune NxT flow cytometer (Thermo Fisher Scientific). Cells negative for both Annexin V and PI were classified as viable, Annexin V-positive/PI-negative cells as early apoptotic, and Annexin V-positive/PI-positive cells as late apoptotic or dead cells.

For cell-cycle analysis, HCT116 cells were seeded and treated under the same conditions. Where indicated, 5 mM NaB was included as a high-concentration comparator. After treatment, floating and adherent cells were collected, washed twice with DPBS, and fixed in 70% ethanol for 1 h. The fixed cells were washed to remove residual ethanol and stained with PI solution for 30 min at room temperature in the dark. DNA content was analyzed using an Attune NxT flow cytometer, and the percentages of cells in the G0/G1, S, and G2/M phases were determined. Experiments were performed independently three times.

### 2.7 Western blot analysis

Total proteins from cultured cells and mouse tumor tissues were extracted using M-PER™ and T-PER™ reagents, respectively (Thermo Fisher Scientific). Protein concentrations were determined using a BCA protein assay kit (Thermo Fisher Scientific). Equal amounts of protein (50 μg per lane) were separated by 13% SDS–polyacrylamide gel electrophoresis and transferred onto nitrocellulose membranes. The membranes were blocked with SuperBlock™ blocking buffer (Thermo Fisher Scientific) and incubated overnight at 4 °C with the indicated primary antibodies at a dilution of 1:1,000. Primary antibodies against histone H3, acetylated histone H3, cleaved caspase-3, cleaved poly(ADP-ribose) polymerase (PARP), phosphorylated p53, and β-actin were purchased from Cell Signaling Technology (MA, USA). Total histone H3 was used to normalize acetylated histone H3 levels, whereas β-actin was used as the loading control for the other target proteins. After primary antibody incubation, the membranes were washed three times with Tris-buffered saline containing 0.1% Tween 20 (TBS-T) and incubated with the appropriate horseradish peroxidase-conjugated secondary antibodies (Cell Signaling Technology) for 1 h at room temperature. Protein bands were visualized using an enhanced chemiluminescence substrate (Thermo Fisher Scientific) and captured using a Fusion Solo S imaging system (Vilber, Île-de-France, France). Band intensities were quantified using ImageJ software, and acetylated histone H3 levels were normalized to total histone H3. The resulting ratios were expressed relative to the RCM control group.

### 2.8 Reverse transcription–quantitative PCR

Total RNA was isolated from HCT116 cells using an RNeasy Mini Kit (QIAGEN, North Rhine-Westphalia, Germany) according to the manufacturer’s instructions. RNA concentration and purity were assessed before cDNA synthesis. cDNA was synthesized from the isolated RNA using a High-Capacity cDNA Reverse Transcription Kit (Thermo Fisher Scientific) according to the manufacturer’s protocol. Quantitative real-time PCR was performed to determine the relative expression levels of *CASP8* and *CASP9*. The primer sequences were as follows: *ACTB*, forward 5′-CACAGAGCCTCGCCTTTG-3′ and reverse 5′-GAATCCTTCTGACCCATGCC-3′; *CASP8*, forward 5′-CTGCTGGGGATGGCCACTGTG-3′ and reverse 5′-TCGCCTCGAGGACATCGCTCTC-3′; and *CASP9*, forward 5′-TGTCTACGGCACAGATGGA-3′ and reverse 5′-GGACTCGTCTTCAGGGGA-3′. Relative gene expression was normalized to *ACTB*. Experiments were performed independently three times.

### 2.9 Whole-genome sequencing and genome analysis

Whole-genome sequencing of *E. limosum* KGMB01548 was performed by DNA Link (Republic of Korea) using the Illumina NovaSeq platform (Illumina, CA, USA). After quality trimming, the paired-end reads were assembled using SPAdes version 3.13.0 (Bankevich et al., 2012). Genome completeness and contamination were evaluated using ContEst16S (Lee et al., 2017) and CheckM (Parks et al., 2015). Coding DNA sequences (CDSs) were predicted using Prodigal (Hyatt et al., 2010). Functional annotation of the predicted CDSs was performed through homology searches against the Swiss-Prot, eggNOG version 4.5 (Huerta-Cepas et al., 2016), SEED, and KEGG databases. tRNA genes were identified using tRNAscan-SE (Lowe and Eddy, 1997), and rRNA genes were identified by covariance model searches against the Rfam version 12.0 database (Nawrocki et al., 2015). Putative genes involved in butyrate biosynthesis were identified by BLAST searches of the assembled genome using reference sequences from previously characterized bacterial butyrate-biosynthesis pathways. The presence and genomic locations of genes predicted to encode thiolase (*thl*), 3-hydroxybutyryl-CoA dehydrogenase (*hbd*), crotonase (*cro*), butyryl-CoA dehydrogenase (*bcd*), electron transfer flavoprotein subunits alpha and beta (*etfA* and *etfB*), and butyryl-CoA:acetate CoA-transferase (*but*) were examined.

### 2.10 Animal experiments and CT26 syngeneic tumor model

A total of 20 male BALB/c mice, 6–8 weeks of age, were purchased from KOSA BIO Inc. (Seongnam, Republic of Korea). The mice were randomly assigned to four experimental groups (*n* = 5 per group) before treatment initiation. No formal allocation-concealment procedure was used, and no formal *a priori* sample-size calculation was performed. For the peritumoral administration experiment, mice were assigned to the RCM control and ElCFS groups. For the oral pre-administration experiment, mice were assigned to the PBS control and live *E. limosum* groups. Mice were housed under specific pathogen-free conditions at 22 ± 2 °C and 55 ± 5% relative humidity with a 12-h light/dark cycle. Standard rodent chow and water were provided *ad libitum*. All animal procedures were approved by the Institutional Animal Care and Use Committee of the Korea Research Institute of Bioscience and Biotechnology (approval no. KRIBB-AEC-20095) and were performed in accordance with the institutional guidelines for the care and use of laboratory animals. Humane endpoints were predefined in the approved animal experimental protocol. The criteria for humane-endpoint euthanasia were tumor-associated ulceration, necrosis, or bleeding; body-weight loss ≥20% relative to the day of CT26 inoculation (day 0) or an absolute body weight ≤17 g; refusal of food or water for more than 24 h; or inability to maintain normal ambulation. No separate analytical exclusion criteria were prespecified, and no animals were excluded from the analyses.

For establishment of the CT26 syngeneic tumor model, CT26 cells were suspended in DPBS at a concentration of 4 × 10^7^ cells/mL. Each mouse was subcutaneously inoculated with 50 μL of the cell suspension, corresponding to 2 × 10^6^ cells, into the right flank. Tumor length and width were measured three times per week using calipers, and tumor volume was calculated using the following equation:


$$\text{Tumor volume}\;({\mathrm{mm}}^{3})=0.5\times \text{length}\times {\text{width}}^{2}$$

Tumor measurements were performed by the same investigator throughout the study using a consistent measurement procedure. Treatment administration, tumor measurement, necropsy, and histological quantification were performed without blinding to group allocation.

All procedures for preparation and enumeration of live *E. limosum* KGMB01548 were performed in an anaerobic chamber. The prepared bacterial suspension and DPBS used for oral administration were kept in a GasPak EZ Anaerobe Pouch System (BD) until immediately before oral gavage to minimize exposure to atmospheric oxygen. For the oral pre-administration experiment, mice received 1 × 10^8^ CFU of live *E. limosum* KGMB01548 suspended in 100 μL of DPBS by oral gavage once daily for 2 weeks before CT26 cell inoculation. Following tumor inoculation, live bacteria were administered once weekly until the end of the experiment. Control mice received an equivalent volume of DPBS according to the same schedule. Necropsy was performed on day 32, when blood, tumor tissues, and spleens were collected for downstream analyses. Fecal samples for 16S rRNA gene amplicon sequencing were collected on the same day.

For the peritumoral ElCFS administration experiment, treatment was initiated 4 days after CT26 cell inoculation and repeated every 3 days. At each administration, 200 μL of ElCFS was divided equally and injected at four sites surrounding the tumor (50 μL per site). The matched control group received an equivalent volume of RCM using the same administration procedure and schedule. Necropsy and tumor collection were performed on day 29. No unscheduled deaths or humane-endpoint euthanasia occurred before scheduled necropsy in either experiment, and all five mice per group completed the study.

### 2.11 Serum butyrate measurement by ELISA

Serum butyrate concentrations were measured using a commercially available butyric acid ELISA kit (Abbexa, Cambridgeshire, UK) according to the manufacturer’s instructions. Briefly, butyrate standards and appropriately diluted mouse serum samples were added to the pre-coated wells and incubated as specified by the manufacturer. After the wells were washed with the supplied wash buffer, tetramethylbenzidine (TMB) substrate was added for color development. The reaction was terminated using the provided stop solution, and absorbance was measured immediately at 450 nm using a microplate reader. Butyrate concentrations were calculated from the standard curve. Each serum sample was analyzed in technical triplicate.

### 2.12 Immunohistochemical staining

Tumor tissues were fixed in neutral-buffered formalin, embedded in paraffin, and sectioned at a thickness of 4 μm. Immunohistochemical staining was performed with minor modifications to the antibody manufacturer’s protocol. Briefly, tissue sections were deparaffinized in xylene, rehydrated through a graded ethanol series, and subjected to heat-induced antigen retrieval in sodium citrate buffer (pH 6.0). The sections were incubated with an anti-CD3 primary antibody (Abcam, Cambridgeshire, UK) at a dilution of 1:100 for more than 6 h at 4 °C. Immunoreactivity was detected using an avidin–biotin-based detection system (Vector Laboratories, CA, USA). The sections were subsequently counterstained with hematoxylin and mounted using an appropriate mounting medium. CD3-positive cells were counted in three non-overlapping fields per tumor at ×400 magnification. The mean number of CD3-positive cells across the three fields was calculated for each mouse and used for statistical analysis.

### 2.13 16S rRNA gene sequencing and gut microbiome analysis

Fecal samples were collected from mice in the CT26 syngeneic tumor experiment, and bacterial community profiles were analyzed by 16S rRNA gene amplicon sequencing. The V3–V4 hypervariable region of the bacterial 16S rRNA gene was amplified using primers 341F (5′-CCTACGGGNGGCWGCAG-3′) and 805R (5′-GACTACHVGGTATCTAATCC-3′) (Herlemann et al., 2011). Amplicons were sequenced on an Illumina MiSeq platform by Macrogen (Seoul, Republic of Korea). Adapter and primer sequences were removed using Cutadapt version 3.2 (Martin, 2011). Raw sequence reads were subsequently processed using DADA2 version 1.18.0 in R (Callahan et al., 2016). Forward and reverse reads were truncated to 250 and 200 bp, respectively, and reads with expected error values ≥2 were removed. Following error-model construction and denoising, paired-end reads were merged, and chimeric sequences were removed using the consensus method to generate amplicon sequence variants (ASVs) (Callahan et al., 2017). After initial quality filtering, 59,848–99,217 reads per sample remained, and after denoising, paired-end merging, chimera removal, and ASV length filtering, 29,200–79,046 reads per sample were retained for downstream analysis. Representative ASV sequences were taxonomically assigned using BLASTN version 2.9.0 + against the NCBI 16S ribosomal RNA sequence database (Camacho et al., 2009). Taxonomic assignments were based on reference sequences showing at least 97% query coverage and sequence identity. Gut microbial community analyses were performed using QIIME version 1.9 (Caporaso et al., 2010) based on the ASV abundance table. To normalize sequencing depth across samples, the ASV abundance table was subsampled to the sequencing depth of the sample with the lowest number of retained reads. Alpha diversity was evaluated using the number of observed ASVs, the Chao1 richness estimator, and the Shannon diversity index. Relative taxonomic abundances at the phylum and finer taxonomic levels were compared between PBS-treated and *E. limosum*-treated mice. Predicted microbial functional profiles were inferred from the 16S rRNA gene sequencing data using PICRUSt2 (Phylogenetic Investigation of Communities by Reconstruction of Unobserved States 2) (Douglas et al., 2020). Predicted abundances of selected KEGG orthologs associated with butyrate-related metabolism were compared between the PBS-treated and *E. limosum*-treated groups. These data represent inferred functional potential rather than directly measured gene abundance or pathway activity.

### 2.14 Statistical analysis

Statistical analyses were performed using GraphPad Prism version 9.0 (GraphPad Software, CA, USA). Data are presented as the mean ± standard deviation (SD), unless otherwise indicated. Comparisons between two independent groups were performed using a two-tailed unpaired Student’s *t*-test. Comparisons among three or more groups were performed using one-way analysis of variance (ANOVA), followed by Tukey’s multiple-comparisons test. For experiments involving two independent variables, two-way ANOVA followed by Šídák’s multiple-comparisons test was used. For longitudinal tumor-growth analyses, tumor volumes measured repeatedly in the same mice over time were analyzed separately for the peritumoral and oral-administration experiments using two-way repeated-measures ANOVA, with treatment as the between-subject factor and time as the within-subject factor. Repeated measurements were matched by mouse, and Šídák’s multiple-comparisons test was used to compare treatment groups at individual time points. Terminal tumor weights were analyzed using a two-tailed unpaired Student’s *t*-test. For animal-derived endpoints, each mouse was treated as one biological replicate. For histological quantification, values obtained from multiple fields within each mouse were averaged to obtain one value per mouse before between-group comparisons. A *p* value < 0.05 was considered statistically significant. Statistical significance is indicated as follows: ns, not significant; *p < 0.05; **p < 0.01; ***p < 0.001; and ****p < 0.0001. The number of biological replicates or animals used in each experiment and the statistical test applied to each quantitative panel are specified in the corresponding figure legend.

## 3 Results

### 3.1 Cell-free supernatant from *Eubacterium limosum* KGMB01548 preferentially suppresses colorectal cancer cell proliferation

Using our previously established screening approach for cell-free supernatants from human gut bacterial isolates (Oh et al., 2021), we identified strain KGMB01548 as an isolate whose culture supernatant exhibited marked antiproliferative activity against CRC cells. Phylogenetic analysis based on the 16S rRNA gene supported the identification of KGMB01548 as *Eubacterium limosum*, whereas scanning electron microscopy revealed its rod-shaped morphology (Supplementary Figure S1). We therefore evaluated the antiproliferative activity of the *E. limosum* KGMB01548 cell-free supernatant (ElCFS). Treatment with 10% (v/v) ElCFS for 72 h markedly reduced crystal violet staining and significantly decreased viable HCT116 cell numbers relative to the reinforced clostridial medium (RCM) control (Figures 1A,B). MTT analysis further demonstrated that the antiproliferative activity of ElCFS increased in a concentration-dependent manner (Figure 1C). At 10% (v/v), ElCFS reduced cell proliferation by more than 50% in both human HCT116 and murine CT26 CRC cells, whereas it showed little detectable activity against non-malignant CCD 841 CoN colonic epithelial cells under the same conditions (Figure 1D). These findings indicate that ElCFS preferentially suppresses CRC cell proliferation and provide the experimental basis for the subsequent characterization of its secreted bioactive metabolite(s).

**Figure 1:**
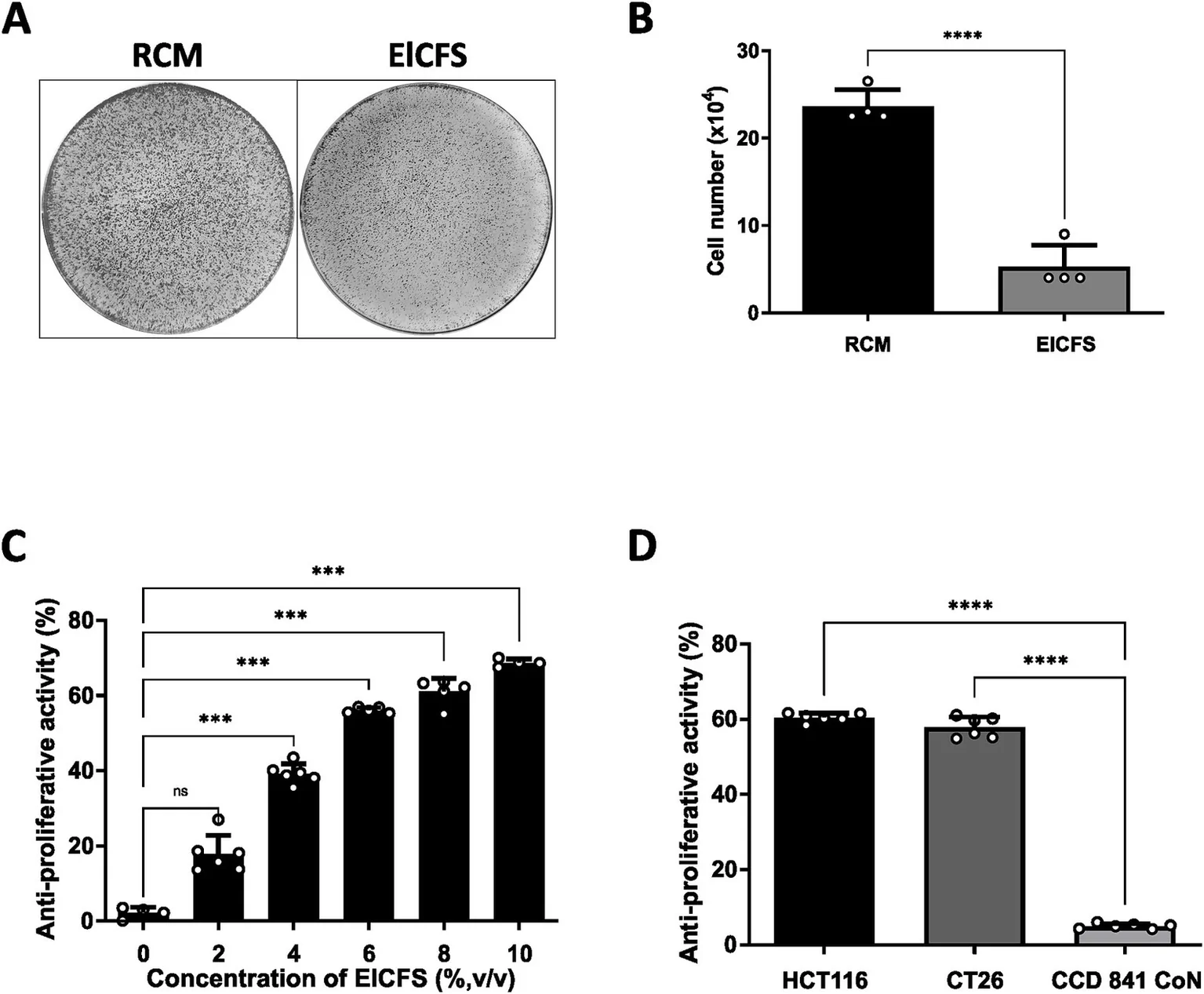
Differential antiproliferative effects of ElCFS on colorectal cancer and non-malignant colonic cells. **(A)** Representative crystal violet staining of HCT116 cells treated with 10% (v/v) RCM or 10% (v/v) ElCFS for 72 h. **(B)** Viable HCT116 cell numbers determined by trypan blue exclusion after treatment with RCM or ElCFS for 72 h. **(C)** Concentration-dependent antiproliferative activity of ElCFS against HCT116 cells, assessed using an MTT assay after 72 h of treatment. **(D)** Antiproliferative activity of 10% (v/v) ElCFS against human HCT116 and murine CT26 colorectal cancer cells and non-malignant human CCD 841 CoN colonic epithelial cells. RCM was used as the matched medium control. Data are presented as the mean ± SD, and individual dots represent independent experiments. Statistical significance in panel **(B)** was assessed using a two-tailed unpaired Student’s *t*-test. Statistical significance in panels **(C)** and **(D)** was assessed using one-way ANOVA followed by Tukey’s multiple-comparisons test. Comparisons were made relative to the RCM control in panel **(B)**, the 0% control in panel **(C)**, and CCD 841 CoN cells in panel **(D)**. ***p < 0.001 and ****p < 0.0001; ns, not significant.

### 3.2 Physicochemical profiling associates ElCFS antiproliferative activity with heat-stable, water-soluble components

To define the physicochemical properties of the ElCFS component(s) associated with growth inhibition, ElCFS was subjected to heat treatment, enzymatic digestion, protein precipitation, and solvent partitioning. Heating ElCFS at 100 °C for 10 min did not appreciably alter its antiproliferative activity against HCT116 cells (Figure 2A), indicating that the activity was heat stable. ElCFS also retained substantial activity after treatment with amylase, papain, trypsin, pepsin, chymotrypsin, proteinase K, or lipase (Figure 2B). These findings suggested that readily degradable proteins, polysaccharides, or lipids were unlikely to be the major contributors to the observed activity. Protein precipitation yielded a protein-enriched fraction with little detectable activity. In a separate experiment, ethyl acetate partitioning of ElCFS at pH 6.8 recovered the activity predominantly in the aqueous fraction, whereas the organic fraction showed little activity (Figure 2C). Collectively, these results indicated that the antiproliferative activity of ElCFS was associated with heat-stable components that resisted the tested enzymatic treatments and preferentially partitioned into the aqueous phase, a physicochemical profile consistent with one or more polar microbial metabolites. This profile prompted subsequent analyses of short-chain fatty acids and the pH-dependent partitioning of the activity.

**Figure 2:**
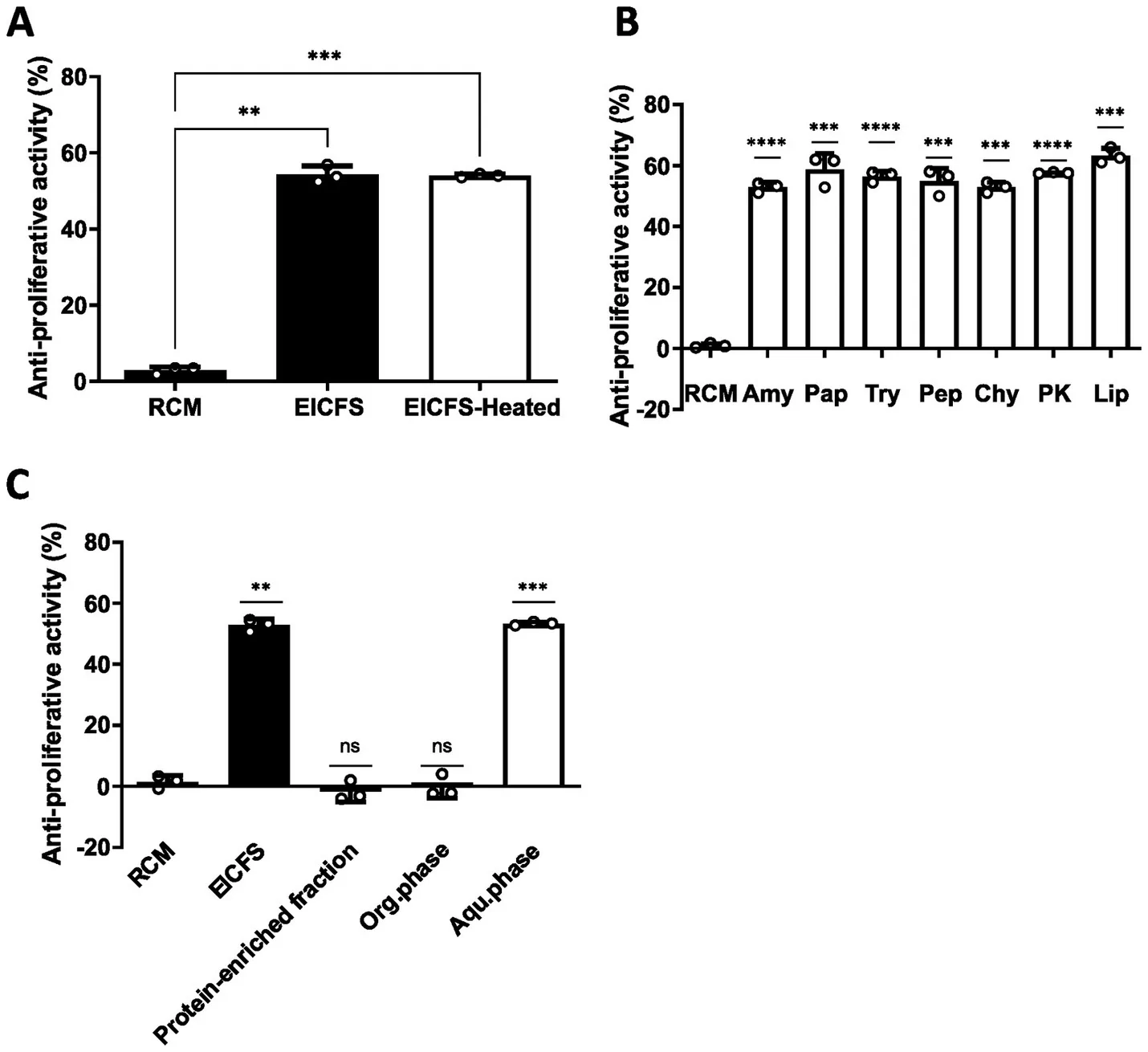
Heat stability, enzyme resistance, and aqueous-phase localization of ElCFS antiproliferative activity. **(A)** Antiproliferative activity of untreated ElCFS and ElCFS heated at 100 °C for 10 min, as determined by the MTT assay after 72 h of treatment. **(B)** Antiproliferative activity of ElCFS after treatment with amylase, papain, trypsin, pepsin, chymotrypsin, proteinase K, or lipase. **(C)** Antiproliferative activity of ElCFS, the protein-enriched fraction, and the organic and aqueous fractions obtained by EtOAc partitioning at pH 6.8. Antiproliferative activity for each treatment or fraction was calculated relative to its corresponding control. The organic fraction was evaporated to dryness and reconstituted in EtOAc before cell treatment. HCT116 cells were used in all experiments. Amy, amylase; Pap, papain; Try, trypsin; Pep, pepsin; Chy, chymotrypsin; PK, proteinase K; Lip, lipase; EtOAc, ethyl acetate; Org.phase, organic-phase fraction; Aqu.phase; aqueous-phase fraction. Data are presented as the mean ± SD, and individual dots represent independent experiments. Statistical significance in panel **(A–C)** was assessed using two-tailed unpaired Student’s *t*-tests, with each treatment or fraction compared with its corresponding RCM control. **p < 0.01, ***p < 0.001, and ****p < 0.0001; ns, not significant.

### 3.3 Integrated physicochemical and functional analyses implicate butyrate as a major candidate contributor to the antiproliferative activity of ElCFS

Because the antiproliferative activity of ElCFS was heat stable, resistant to the tested enzymatic treatments, and predominantly recovered in the aqueous fraction at neutral pH, we next evaluated whether short-chain fatty acids contributed to this activity. High-performance liquid chromatography analysis detected acetate and butyrate as the major quantified SCFAs in ElCFS, at concentrations of 23.33 and 13.77 mM, respectively (Table 1). We next examined whether the pH-dependent redistribution of ElCFS activity was consistent with SCFA partitioning. As weak acids with pKa values of approximately 4.8, SCFAs remain largely in the aqueous phase at neutral pH but preferentially partition into the organic phase after acidification, a principle commonly used for SCFA extraction (Kim et al., 2022). A similar acidification-dependent decrease in aqueous-phase butyrate was previously reported for *Eubacterium callanderi* CFS (Ryu et al., 2022). Consistent with these physicochemical properties, antiproliferative activity was recovered predominantly in the aqueous fraction at pH 6.8, whereas acidification to pH 2.5 shifted most of the activity to the ethyl acetate fraction (Figure 3A). The concentration of butyrate remaining in the aqueous fraction decreased from 12.47 mM at pH 6.8 to 0.32 mM at pH 2.5, whereas the aqueous acetate concentration decreased from 23.04 to 10.28 mM (Table 1). The close correspondence between the marked depletion of aqueous-phase butyrate and the redistribution of antiproliferative activity to the organic fraction provided a rationale for the subsequent functional evaluation of the major SCFAs detected in ElCFS. NaB inhibited HCT116 cell proliferation in a concentration-dependent manner over the tested range of 0.5–2 mM, whereas sodium acetate at 1–5 mM showed little detectable activity (Supplementary Figure S2). Because treatment with 10% (v/v) ElCFS was expected to contribute 13.77 mM × 0.10 = 1.377 mM, approximately 1.38 mM, butyrate to the final culture medium, we compared ElCFS with 1.3 mM NaB as an approximately concentration-matched comparator. ElCFS and approximately concentration-matched NaB produced comparable levels of antiproliferative activity in HCT116 cells (Figure 3B). Both treatments also increased histone H3 acetylation relative to the control groups (Figure 3C), a response consistent with the established HDAC-inhibitory activity of butyrate. To determine whether ElCFS reproduced the glucose-dependent activity previously described for low-dose butyrate, HCT116 cells were treated with ElCFS or 1.3 mM NaB under glucose-containing and glucose-depleted conditions (Donohoe et al., 2012). The MTT-based inhibitory effects of both ElCFS and 1.3 mM NaB were markedly attenuated under glucose-depleted conditions compared with glucose-containing conditions (Figure 3D). In contrast, the inhibitory activity of 5 mM NaB was largely retained under glucose-depleted conditions (Supplementary Figure S3). To complement these physicochemical and functional findings with strain-level genomic evidence, we analyzed the genome of *E. limosum* KGMB01548. Whole-genome sequencing yielded a 4,571,659 bp draft genome comprising 34 contigs and 4,337 predicted coding sequences (Supplementary Tables S1, S2). Genome annotation identified genes predicted to encode the acetyl-CoA-to-butyrate pathway, including thiolase, 3-hydroxybutyryl-CoA dehydrogenase, crotonase, butyryl-CoA dehydrogenase/electron transfer flavoproteins, and the terminal enzyme butyryl-CoA:acetate CoA-transferase. These findings support the genetic capacity of KGMB01548 to synthesize butyrate (Supplementary Figure S4) (Vital et al., 2014; Jeong et al., 2015). Collectively, the SCFA composition of ElCFS, pH-dependent redistribution of antiproliferative activity, comparable effects of approximately concentration-matched NaB, increased histone H3 acetylation, glucose dependence, and genomic evidence for butyrate biosynthesis support butyrate as a major candidate contributor to the antiproliferative activity of ElCFS. However, because ElCFS contains a complex mixture of microbial metabolites, these findings do not establish that butyrate is necessary for its activity or quantify its contribution. Other metabolites, alone or in combination with butyrate, may also contribute.

**Table 1:** Concentrations of acetate and butyrate in *Eubacterium limosum* cell-free supernatant and aqueous fractions obtained after pH-dependent liquid–liquid extraction.

Short-chain fatty acids (SCFA)	Concentration (mM)		
	CFS	pH 6.8 Aqueous phase	pH 2.5 Aqueous phase
Acetic acid	23.33	23.04	10.28
Butyric acid	13.77	12.47	0.32

**Figure 3:**
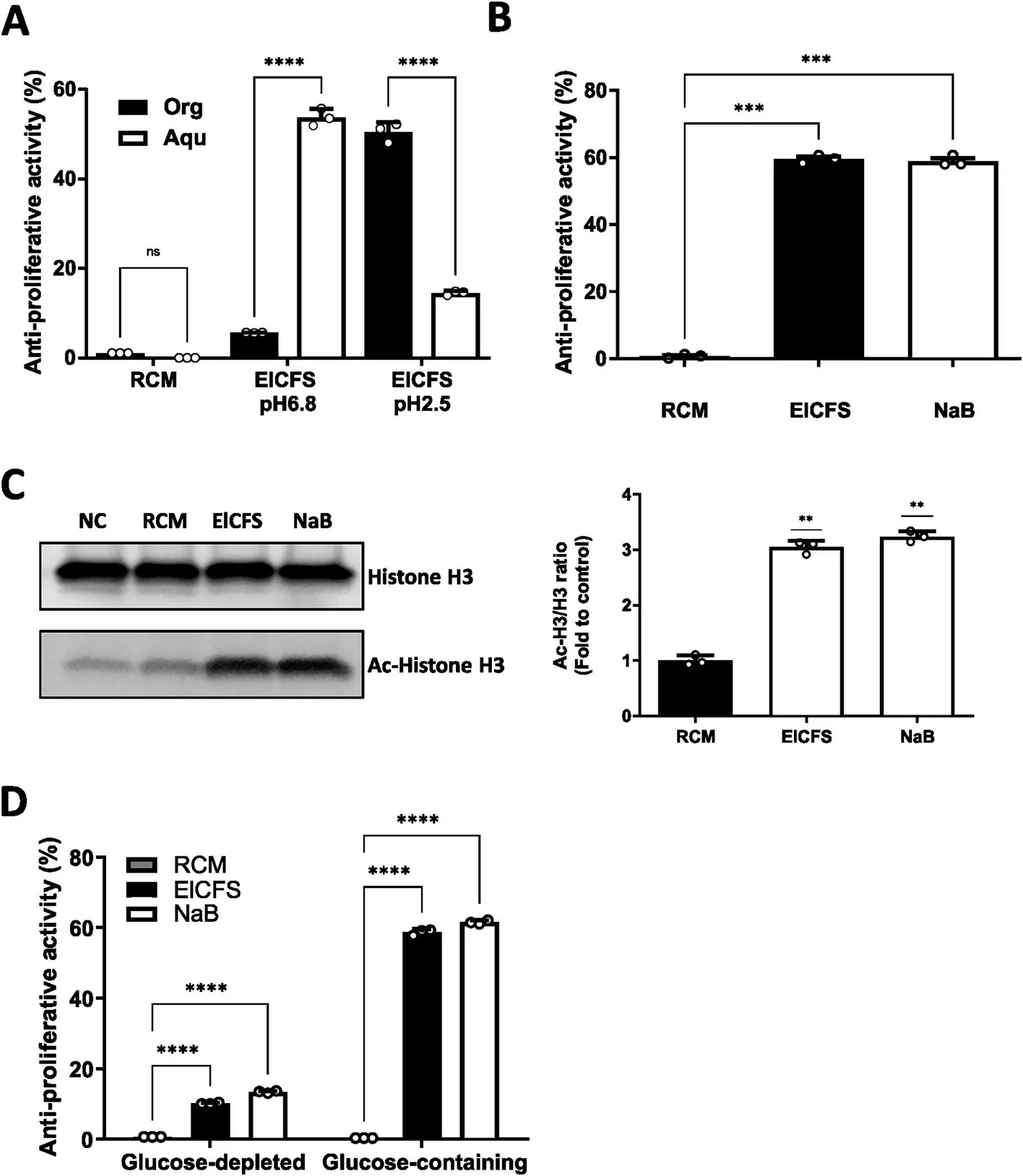
Physicochemical and cellular responses linking butyrate to ElCFS antiproliferative activity. **(A)** Antiproliferative activity of the aqueous and ethyl acetate fractions obtained by liquid–liquid extraction of ElCFS at pH 6.8 and pH 2.5. Antiproliferative activity was calculated relative to the corresponding fractions prepared from RCM under the same extraction. **(B)** Comparison of the antiproliferative activities of 10% (v/v) ElCFS and approximately concentration-matched sodium butyrate (NaB; 1.3 mM) in HCT116 cells. **(C)** Representative western blot images of acetylated histone H3 (Ac-H3) and total histone H3, together with densitometric quantification of the Ac-H3/H3 ratio normalized to the RCM. (D) MTT-based antiproliferative activities of ElCFS and 1.3 mM NaB under glucose-containing and glucose-depleted culture conditions. In panels **(B)** and **(D)**, antiproliferative activity was calculated relative to the matched RCM control under each culture condition. Org, organic-phase; Aqu, aqueous-phase; Ac-H3, acetylated histone H3; NaB, sodium butyrate. Data are presented as the mean ± SD, and individual dots represent independent experiments. Statistical significance was assessed using two-way ANOVA followed by Šídák’s multiple-comparisons test in panels **(A)** and **(D)**, and one-way ANOVA followed by Tukey’s multiple-comparisons test in panels **(B)** and **(C)**. In panel **(A)**, the organic and aqueous fractions were compared within each extraction condition. In panels **(B–D)**, comparisons were made relative to the corresponding RCM control. **p < 0.01, ***p < 0.001, and ****p < 0.0001; ns, not significant.

### 3.4 ElCFS and concentration-matched butyrate induce apoptosis with limited changes in cell-cycle distribution in HCT116 cells

Having found that ElCFS and approximately concentration-matched NaB exerted comparable antiproliferative and histone-acetylating effects, we next examined whether growth inhibition was associated with apoptosis or changes in cell-cycle distribution. Annexin V-FITC/propidium iodide (PI) flow cytometry showed an increased Annexin V-positive population following treatment with either ElCFS or 1.3 mM NaB (Figure 4A) (Pucci et al., 2000). In the representative profiles, the total apoptotic fraction, calculated as the sum of early and late apoptotic cells, was 17.73% in the ElCFS group and 17.17% in the NaB group, compared with 1.76% in the RCM group and 2.20% in the untreated control group, respectively. Cell-cycle analysis showed only modest changes following treatment with ElCFS or 1.3 mM NaB. ElCFS increased the G0/G1-phase fraction and reduced the S-phase fraction, whereas approximately concentration-matched NaB produced smaller changes in cell-cycle distribution (Figure 4B). These effects were less pronounced than the marked cell-cycle redistribution observed with 5 mM NaB, which increased the G2/M-phase fraction (Supplementary Figure S5). Consistent with the flow-cytometric findings, western blot analysis showed increased levels of cleaved PARP and cleaved caspase-3 in ElCFS- and NaB-treated cells, together with increased p53 phosphorylation (Figure 4C) (Vaseva and Moll, 2009; Chaitanya et al., 2010). The response pattern induced by ElCFS was broadly similar to that observed with approximately concentration-matched NaB. Because butyrate has been associated with mitochondrial apoptotic signaling in CRC cells, we further examined *CASP8* and *CASP9* mRNA expression by quantitative reverse-transcription PCR (RT-qPCR) (Fung et al., 2012). *CASP9* expression was significantly higher in the ElCFS- and NaB-treated groups than in the RCM control group, whereas *CASP8* expression was not significantly altered (Figure 4D). This expression pattern is consistent with preferential engagement of intrinsic apoptotic signaling, although pathway dependence was not directly established. Collectively, these findings indicate that, under the conditions tested, ElCFS phenocopied concentration-matched NaB by inducing apoptosis, whereas its effects on cell-cycle distribution were comparatively limited.

**Figure 4:**
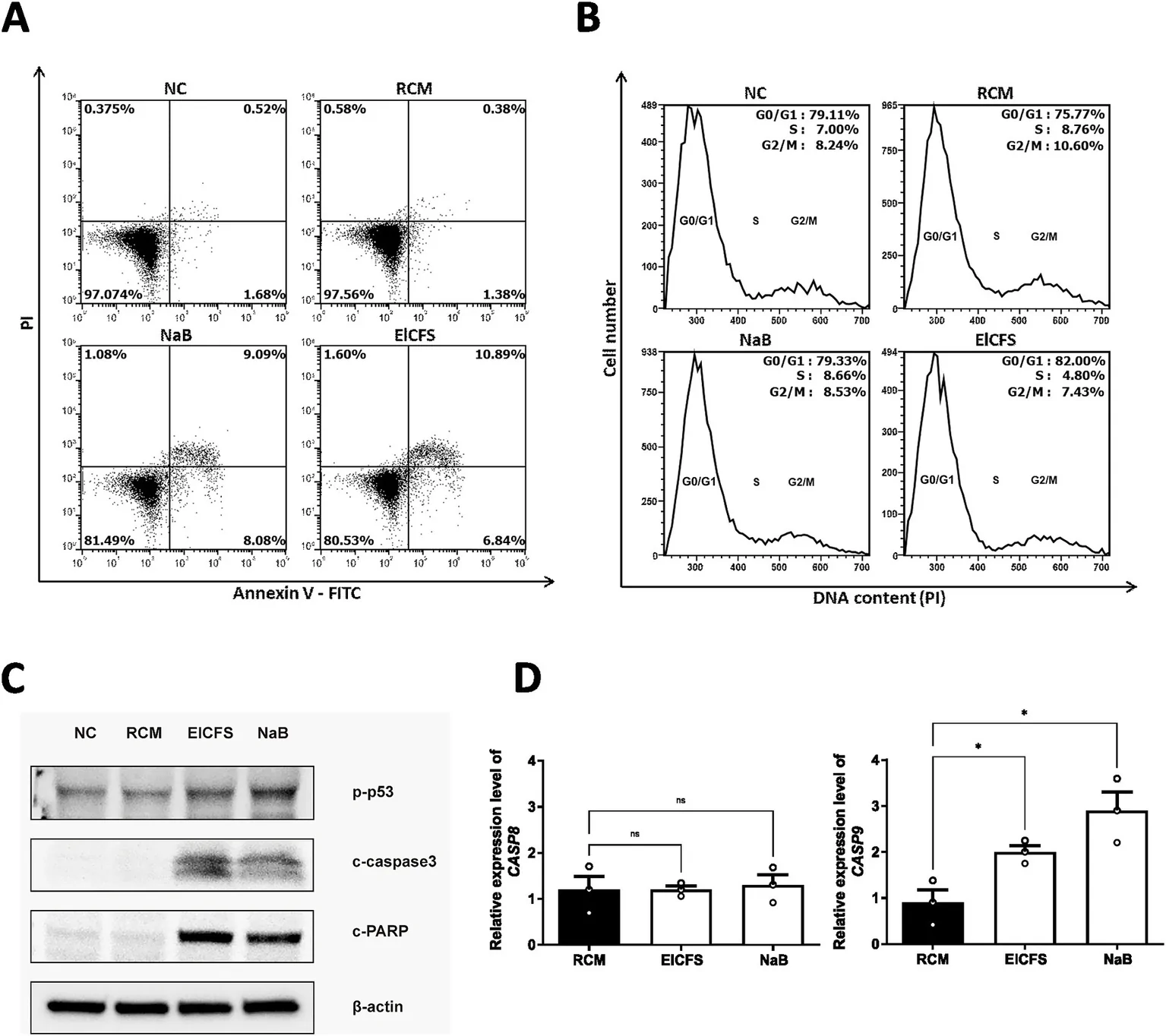
Apoptotic responses and cell-cycle profiles induced by ElCFS and approximately concentration-matched NaB. **(A)** Representative flow-cytometric analysis of apoptosis following treatment with ElCFS or 1.3 mM NaB. Cells were stained with Annexin V-FITC and propidium iodide (PI). **(B)** Representative flow-cytometric analysis of cell-cycle distribution following treatment with ElCFS or 1.3 mM NaB. Cellular DNA was stained with PI. **(C)** Representative western blot analysis of phosphorylated p53, cleaved caspase-3, and cleaved PARP. β-actin was used as the loading control. **(D)** RT-qPCR analysis of *CASP8* and *CASP9* mRNA expression 72 h after treatment. *ACTB* was used as the reference gene. HCT116 cells were used in all experiments. p-p53, phosphorylated p53; c-caspase-3, cleaved caspase-3; c-PARP, cleaved poly(ADP-ribose) polymerase; RCM, reinforced clostridial medium treated control; NaB, sodium butyrate. Quantitative data are presented as the mean ± SD, and individual dots represent independent experiments. Statistical analysis of the quantitative data in panel **(D)** was performed using one-way ANOVA followed by Tukey’s multiple-comparisons test, with comparisons made relative to the RCM control. *p < 0.05; ns, not significant.

### 3.5 Peritumoral ElCFS and oral pre-administration of live *Eubacterium limosum* KGMB01548 reduce tumor burden in a CT26 syngeneic model

To determine whether the antitumor activity observed *in vitro* was retained *in vivo*, we used a CT26 syngeneic subcutaneous tumor model in BALB/c mice (Wu et al., 2011). ElCFS was administered peritumorally beginning 4 days after CT26 cell inoculation. The experimental timelines are shown in Figure 5A. At the final measurement, the mean tumor volume was 52.62% (1272.20 ± 177.58 vs. 602.72 ± 173.43 mm^3^) lower in the ElCFS-treated group than in the matched RCM control group, and terminal tumor weight was reduced by 37.83% (2.18 ± 0.58 vs. 1.35 ± 0.04 g) (Figures 5B,C). Representative images of the excised tumors were consistent with these quantitative findings (Figure 5D). In a separate experiment, mice received live *E. limosum* KGMB01548 by oral gavage daily for 2 weeks before CT26 cell inoculation and weekly thereafter. Compared with the PBS-treated control group, oral pre-administration of *E. limosum* reduced final tumor volume by 44.83% (2049.89 ± 184.11 vs. 1130.84 ± 311.49 mm^3^) and terminal tumor weight by 32.53% (3.19 ± 0.13 vs. 2.15 ± 0.13 g) (Figures 5E,F). Representative images of the excised tumors showed the same overall trend (Figure 5G). We next examined whether oral administration of *E. limosum* was accompanied by changes in circulating butyrate and tumor-associated host responses. Serum butyrate concentrations were numerically higher in the *E. limosum*-treated group than in the PBS-treated group, although the difference was not statistically significant (Figure 6A). Exploratory flow-cytometric profiles of splenic CD3-positive T cells suggested an increased proportion of CD4-positive cells following oral administration of *E. limosum* (Supplementary Figure S6). Immunohistochemical analysis further showed a higher number of CD3-positive cells in tumor sections from mice orally administered *E. limosum* than in those from PBS-treated mice (Figure 6B) (Brummel et al., 2023). Representative immunoblots also showed stronger cleaved caspase-3 signals in tumors from both the oral *E. limosum* group and the peritumoral ElCFS group than in their respective control groups (Figure 6C). Overall, peritumoral administration of ElCFS and oral pre-administration of live *E. limosum* KGMB01548 reduced tumor burden in the CT26 syngeneic model. In the oral-administration experiment, reduced tumor burden was accompanied by an increased number of tumor-associated CD3-positive cells, whereas cleaved caspase-3 signals were enhanced in tumors from both intervention groups. These associations suggest treatment-related host and apoptotic responses but do not establish that circulating butyrate or systemic T-cell changes directly mediated tumor suppression.

**Figure 5:**
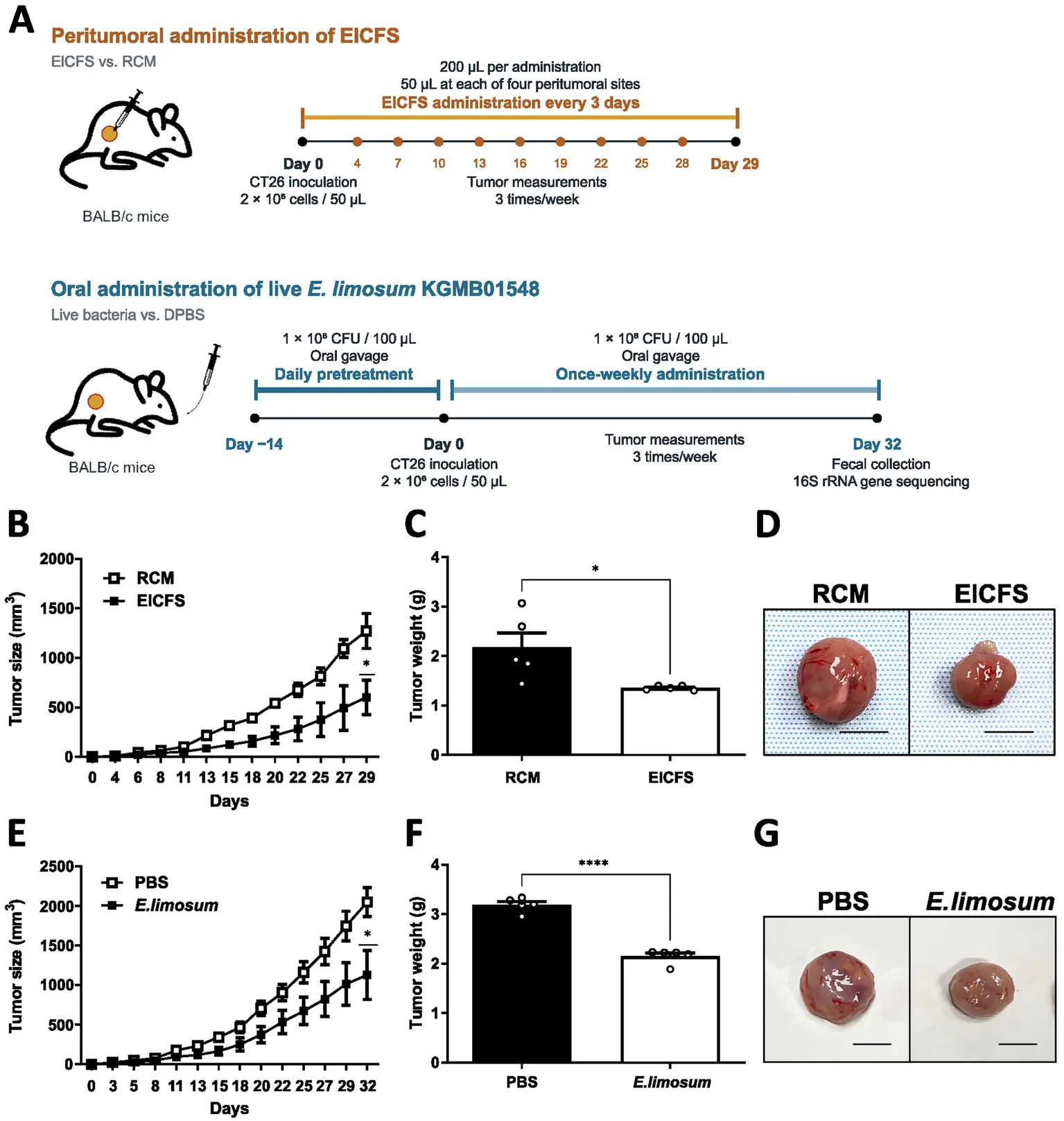
Tumor growth responses to peritumoral ElCFS delivery and oral pre-administration of live *Eubacterium limosum*. **(A)** Experimental scheme of the CT26 syngeneic tumor model. **(B–D)** Peritumoral ElCFS administration experiment. RCM was used as the matched control. **(E–G)** Oral pre-administration experiment using live *E. limosum* KGMB01548. PBS was used as the control. **(B,E)** Tumor-volume curves for the peritumoral ElCFS experiment and the oral *E. limosum* experiment. **(C,F)** Terminal tumor weights measured at necropsy. **(D,G)** Representative images of excised tumors collected at necropsy. Data are presented as the mean ± SD, and individual dots represent individual mice (*n* = 5 mice per group). Tumor-volume curves in panels **(B)** and **(E)** were analyzed using two-way repeated-measures ANOVA, with treatment as the between-subject factor and time as the within-subject repeated factor, followed by Šídák’s multiple-comparisons test. Terminal tumor weights in panels **(C)** and **(F)** were analyzed using a two-tailed unpaired Student’s *t*-test. Comparisons were made between the RCM and ElCFS groups in panels **(B)** and **(C)**, and between the PBS and *E. limosum* groups in panels **(E)** and **(F)**. *p < 0.05 and ****p < 0.0001. Scale bar, 1 cm.

**Figure 6:**
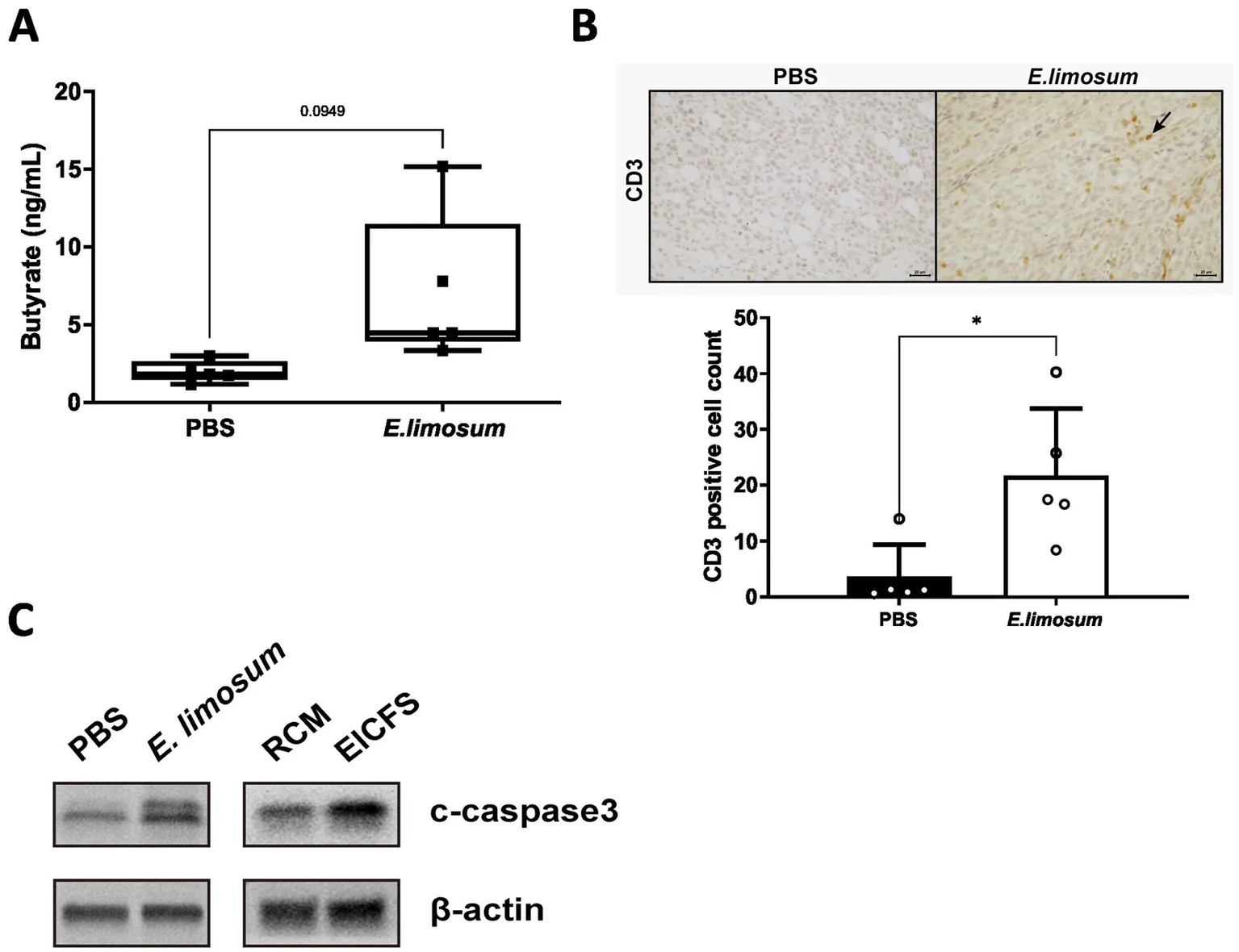
Circulating butyrate and tumor-associated host responses following *Eubacterium limosum* or ElCFS administration. **(A)** Serum butyrate concentrations in mice orally administered PBS or live *Eubacterium limosum* KGMB01548. **(B)** Representative immunohistochemical images and quantitative analysis of CD3-positive cells in tumor tissues from the oral-administration experiment. Brown-stained cells indicate CD3-positive cells, and arrows indicate representative positive cells. **(C)** Representative western blot analysis of cleaved caspase-3 in tumor tissues from the oral *E. limosum* experiment (left) and the peritumoral ElCFS experiment (right). β-Actin was used as the loading control. Data are presented as the mean ± SD, and individual dots represent individual mice (*n* = 5 mice per group). c-caspase-3, cleaved caspase-3; RCM, reinforced clostridial medium. Statistical significance in panels **(A)** and **(B)** was assessed using a two-tailed unpaired Student’s *t*-test. Comparisons were made relative to the PBS treated group. *p < 0.05.

### 3.6 Oral administration of *Eubacterium limosum* KGMB01548 is associated with modest gut microbial and predicted functional shifts

To examine whether oral administration of live *Eubacterium limosum* KGMB01548 was associated with changes in the gut microbial community, we performed 16S rRNA gene amplicon sequencing of mouse fecal samples. The number of observed ASVs, together with the Chao1 richness estimator and Shannon diversity index, did not differ significantly between the PBS-treated and *E. limosum*-treated groups (Figure 7A) (Chao, 1987; Callahan et al., 2017; Willis, 2019; Magurran, 2021). These findings indicated that oral administration of *E. limosum* did not produce a major change in within-sample microbial richness or diversity. Phylum-level microbial profiles were also broadly similar between the two groups (Figure 7B). The *Bacillota/Bacteroidota* ratio was lower in the *E. limosum*-treated group than in the control group (Figure 7C). At the individual phylum level, *Actinomycetota*, *Deferribacterota*, and *Pseudomonadota* showed numerically lower mean relative abundances in the *E. limosum*-treated group than in the control group, although none of these differences reached statistical significance (Supplementary Figure S7). At finer taxonomic resolution, several taxa previously associated with short-chain fatty acid production displayed higher relative abundances following *E. limosum* administration (Figure 8A). The relative abundance of *Eubacterium* spp. also showed a modest increase in the treated group (Figure 8B). Functional inference using PICRUSt2 further suggested slightly higher predicted abundances of selected orthologs associated with butyrate-related metabolic pathways in the *E. limosum*-treated group (Figure 8C). Because these functional profiles were inferred from 16S rRNA gene data, they represent predicted metabolic potential rather than directly measured gene abundance or pathway activity. Overall, oral administration of *E. limosum* KGMB01548 was associated with modest taxonomic and predicted functional shifts without a major alteration in overall gut microbial diversity.

**Figure 7:**
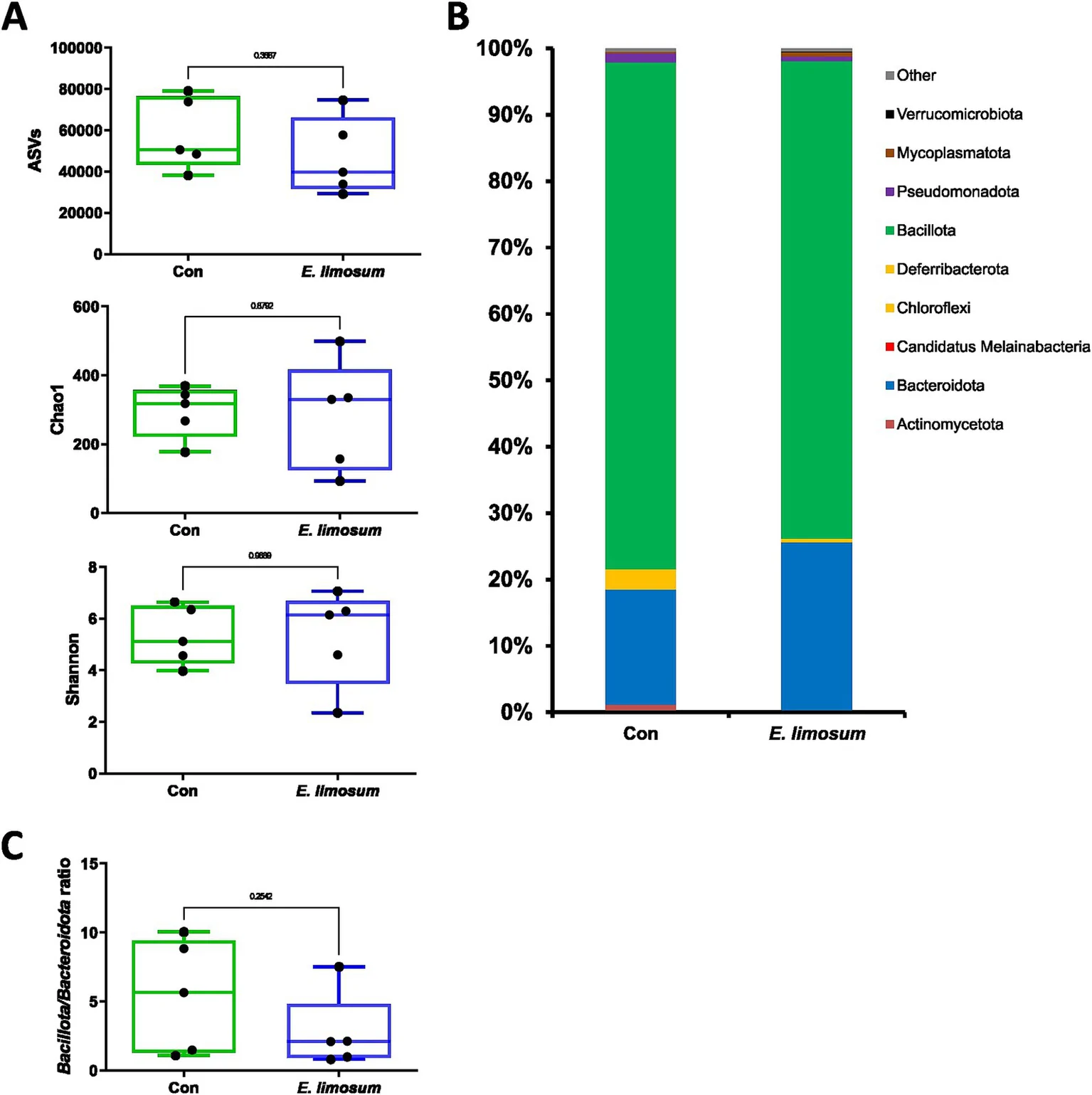
Gut microbial diversity and phylum-level composition following oral administration of *Eubacterium limosum*. **(A)** Comparison of within-sample microbial richness and diversity between PBS-treated and *E. limosum*-treated mice, based on the number of observed amplicon sequence variants (ASVs), the Chao1 richness estimator, and the Shannon diversity index. **(B)** Relative abundance of gut bacterial taxa at the phylum level. **(C)** Comparison of the *Bacillota/Bacteroidota* ratio between the two groups. Con; PBS-treated control group; *E. limosum*; *Eubacterium limosum* treated group. Data in panels **(A)** and **(C)** are presented as box-and-whisker plots. Statistical significance in panels **(A)** and **(C)** were assessed using a two-tailed unpaired Student’s *t*-test. Comparisons were made relative to the control group (*n* = 5 mice per group).

**Figure 8:**
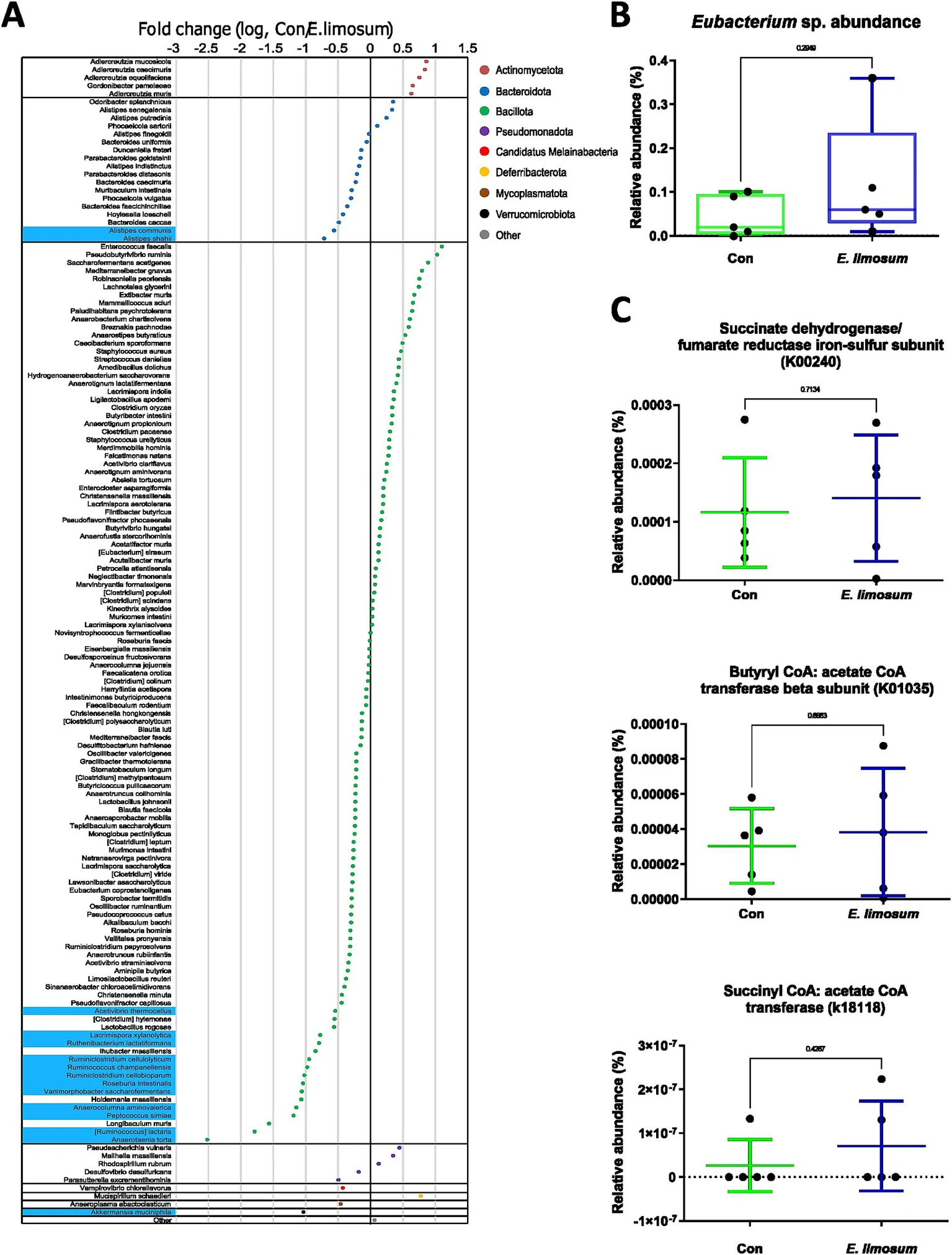
SCFA-associated bacterial taxa and predicted butyrate-related functions following oral administration of *Eubacterium limosum*. **(A)** Fold-change comparison of bacterial taxa between PBS-treated and *E. limosum*-treated mice. Highlighted boxes indicate taxa previously associated with short-chain fatty acid production that displayed higher relative abundance in the control group or the *E. limosum*-treated group. **(B)** Relative abundance of *Eubacterium* spp. in the two groups. **(C)** PICRUSt2-predicted relative abundances of selected KEGG orthologs associated with butyrate-related metabolism. The predicted functional profiles represent inferred metabolic potential based on 16S rRNA gene data and not directly measured gene abundance or pathway activity. Con; PBS-treated control group; *E. limosum*; *Eubacterium limosum* treated group. Data in panel **(B)** are presented as a box-and-whisker plot, whereas data in panel **(C)** are presented as the mean ± SD with individual values shown. Statistical significance in panels **(B)** and **(C)** was assessed using a two-tailed unpaired Student’s *t*-test. Comparisons were made relative to the control group (*n* = 5 mice per group).

## 4 Discussion

The gut microbiota influences CRC through microbial community structure, host–microbe interactions, and the production of bioactive metabolites (Hemarajata and Versalovic, 2013; Ji and Nielsen, 2015; Valdes et al., 2018; Alvarez et al., 2021; Wang et al., 2021). These findings have stimulated interest in gut commensals as potential next-generation probiotic candidates and as sources of microbiome-derived therapeutic molecules (Almeida et al., 2020; Kazmierczak-Siedlecka et al., 2022; Zhang et al., 2022; Sionek et al., 2023; Vallianou et al., 2023). *Eubacterium limosum* is an anaerobic human gut bacterium capable of producing acetate and butyrate and has previously been associated with beneficial effects on intestinal inflammation and mucosal integrity (Kanauchi et al., 2006; Mukherjee et al., 2020). In the present study, we investigated *E. limosum* KGMB01548, an independent human-derived strain, and examined the relationship between its cell-free culture supernatant, butyrate production, CRC cell responses, and tumor suppression. Our results identify butyrate as a major candidate contributor to the *in vitro* antiproliferative activity of ElCFS and show that ElCFS and sodium butyrate at an approximately matched concentration elicit similar histone-acetylation and apoptotic responses. These observations support an association but do not establish a butyrate-dependent mechanism.

A recent study by Lu et al. demonstrated that *E. limosum* El1405 suppressed CRC cell proliferation and tumor growth through a broad combination of mechanisms involving multiple metabolites, including indole-3-lactic acid, indole-3-acetic acid, L-arginine, GABA, and butyrate, together with changes in the intestinal and intratumoral microbiota and the tumor microenvironment (Lu et al., 2025). That study established an important multi-metabolite and ecosystem-level framework for the anti-CRC activity of *E. limosum*. The present study addresses a narrower and complementary mechanistic question using a distinct human-derived strain. Rather than broadly profiling the metabolome and tumor immune microenvironment, we evaluated whether butyrate at the concentration actually present in ElCFS could account for key physicochemical and cellular features of its antiproliferative activity. This distinction is important because the detection of butyrate in a complex bacterial supernatant does not, by itself, establish the extent to which it contributes to the observed biological effect.

ElCFS from strain KGMB01548 preferentially inhibited the proliferation of human HCT116 and murine CT26 CRC cells while showing little detectable activity against non-malignant CCD 841 CoN colonic cells under the same conditions. Similar CRC-selective effects have been reported for the culture supernatant of *E. limosum* El1405 (Lu et al., 2025). Thus, the principal significance of this result is not the initial observation of antiproliferative activity alone, but the extension of this phenotype to an independently isolated strain and the subsequent physicochemical and functional characterization of its secreted metabolites. The activity of ElCFS was retained after heating and treatment with the tested proteolytic, amylolytic, and lipolytic enzymes, and was predominantly recovered in the aqueous fraction at neutral pH. These findings are consistent with the involvement of heat-stable, polar, low-molecular-weight microbial metabolites rather than readily degradable macromolecules.

HPLC analysis detected acetate and butyrate in ElCFS at concentrations of 23.33 and 13.77 mM, respectively. The pH-dependent redistribution of ElCFS activity provided additional physicochemical evidence linking an organic acid to the antiproliferative phenotype. At pH 6.8, the activity remained predominantly in the aqueous fraction, whereas acidification to pH 2.5 shifted most of the activity to the ethyl acetate fraction. This redistribution was accompanied by a marked decrease in aqueous-phase butyrate from 12.47 to 0.32 mM, while a larger proportion of acetate remained in the aqueous phase. This behavior is consistent with the acid–base properties of SCFAs and with previous observations using bacterial culture supernatants (Kim et al., 2022; Ryu et al., 2022). Nevertheless, pH-dependent solvent partitioning is not specific to butyrate, and this result alone cannot identify the active compound.

The functional comparison of the major SCFAs detected in ElCFS provided a more direct link to butyrate. NaB inhibited HCT116 cell proliferation in a concentration-dependent manner, whereas sodium acetate showed little detectable activity over the tested concentration range. Importantly, treatment with 10% ElCFS was calculated to deliver approximately 1.38 mM butyrate to the culture medium, and 1.3 mM NaB reproduced a level of antiproliferative activity comparable to that of ElCFS. This concentration-matched comparison differentiates the present study from approaches that identify bacterial metabolites without testing them at the concentrations actually present in the biologically active supernatant. Genome annotation further identified genes predicted to encode the acetyl-CoA-to-butyrate pathway, including butyryl-CoA:acetate CoA-transferase, thereby supporting the genetic capacity of KGMB01548 to synthesize butyrate (Vital et al., 2014; Jeong et al., 2015). Genomic evidence does not identify the active molecule by itself, but it independently supports the microbial origin of the butyrate quantified in ElCFS.

Butyrate can accumulate in glycolytic CRC cells and inhibit histone deacetylases, resulting in increased histone acetylation and altered expression of genes involved in proliferation, cell-cycle regulation, and apoptosis (Fung et al., 2011; Donohoe et al., 2012; Tan et al., 2014; Xiao et al., 2018; Xi et al., 2021; Ali et al., 2022; Hodgkinson et al., 2023). In the present study, both ElCFS and approximately concentration-matched NaB increased histone H3 acetylation. This shared response is consistent with HDAC inhibition, although HDAC enzyme activity was not directly measured. The antiproliferative effects of both treatments were also markedly attenuated under glucose-depleted conditions, whereas the activity of 5 mM NaB was largely retained. These findings are consistent with the concentration- and metabolic-context-dependent actions of butyrate described in CRC cells (Donohoe et al., 2012). Under highly glycolytic conditions, reduced butyrate oxidation may promote intracellular accumulation and HDAC-inhibitory activity, whereas altered glucose availability can change butyrate utilization and its cellular effects. However, because the glucose-dependence experiment was evaluated using an MTT-based assay, changes in cellular redox metabolism cannot be fully distinguished from changes in viable cell number. Confirmation using a metabolism-independent cell-counting method would further strengthen this interpretation.

ElCFS and 1.3 mM NaB also produced broadly similar apoptotic responses. Both treatments increased the Annexin V-positive population and were associated with PARP and caspase-3 cleavage and increased p53 phosphorylation. In addition, *CASP9* expression increased, whereas *CASP8* expression showed no comparable change. This pattern is consistent with preferential engagement of intrinsic apoptotic signaling, but pathway dependence was not directly demonstrated because mitochondrial membrane potential, cytochrome c release, cleaved caspase-9, or inhibitor-based rescue experiments were not performed (Pucci et al., 2000; Vaseva and Moll, 2009; Chaitanya et al., 2010; Fung et al., 2012). ElCFS and approximately concentration-matched butyrate produced only limited changes in cell-cycle distribution compared with the pronounced redistribution observed at 5 mM NaB. Lu et al. reported both apoptosis and cell-cycle arrest following treatment with El1405CS. The apparent difference between the studies may reflect strain-specific metabolite composition, the CRC cell line examined, treatment duration, or the effective butyrate concentration. Our findings suggest that, at the butyrate concentration present in 10% ElCFS, apoptosis was a more prominent cellular response than marked cell-cycle arrest.

The *in vivo* findings further support the antitumor potential of KGMB01548 and its secreted metabolites. Peritumoral administration of ElCFS beginning after CT26 inoculation reduced tumor volume and terminal tumor weight. Previous work showed that orally administered El1405 culture supernatant suppressed tumor growth (Lu et al., 2025). In contrast, the present local-delivery experiment demonstrates that the secreted components of KGMB01548 retained tumor-suppressive activity when administered peritumorally rather than through the gastrointestinal tract. This finding reduces, but does not completely eliminate, the contribution of intestinal microbial remodeling to the observed effect and provides complementary evidence for local activity of ElCFS. Increased cleaved caspase-3 signals in tumors from ElCFS-treated mice were also consistent with the apoptosis observed *in vitro*. Nevertheless, because purified NaB was not evaluated using the same peritumoral regimen and butyrate-depleted ElCFS was not tested, the *in vivo* effect cannot be attributed exclusively to butyrate. Additional metabolites or interactions among ElCFS components may also have contributed.

Oral pre-administration of live *E. limosum* KGMB01548 also reduced subsequent CT26 tumor burden. Because bacterial administration began 2 weeks before tumor inoculation, this experiment primarily represents a preconditioning or preventive intervention rather than treatment of an established tumor. Tumor sections from mice administered live *E. limosum* contained more CD3-positive cells, and exploratory splenic flow-cytometric profiles suggested a higher proportion of CD4-positive cells within the CD3-positive population. SCFAs can affect both effector and regulatory T-cell differentiation, and CD4-positive T cells may exert either antitumor or immunoregulatory functions depending on their phenotype and context (Park et al., 2015; Luu et al., 2021; Overacre-Delgoffe et al., 2021; Tay et al., 2021; Trend et al., 2021). Therefore, an increase in total CD3- or CD4-positive cells cannot by itself establish functional antitumor T-cell activation. Moreover, serum butyrate concentrations were numerically higher but not significantly different between the treatment groups. These findings indicate that tumor suppression was accompanied by host T-cell-associated changes, but they do not demonstrate that circulating butyrate or systemic T-cell modulation mediated the antitumor response. Functional characterization of tumor-associated CD4, CD8, FoxP3, IFN-γ, or granzyme B-positive populations would be needed to define the immune contribution more precisely.

The gut microbial analysis revealed a different pattern from the extensive community remodeling reported for El1405 (Lu et al., 2025). Oral administration of KGMB01548 did not markedly alter ASV richness, Chao1 estimates, or Shannon diversity, and the overall phylum-level profiles remained broadly similar between groups. Instead, more limited changes were observed in the relative abundances of selected SCFA-associated taxa and *Eubacterium* spp. The *Bacillota/Bacteroidota* ratio was examined because the historically termed *Firmicutes/Bacteroidetes* ratio has been used as a broad index of gut microbial balance in studies of colorectal cancer and intestinal dysbiosis, including experimental CRC models (Yang et al., 2019; Wang et al., 2024). However, its biological significance is context dependent, and the ratio should therefore be interpreted as an exploratory ecological measure rather than a validated marker of CRC or intestinal inflammation. The *Bacillota/Bacteroidota* ratio was also lower in the treated group, but this ratio is a broad and context-dependent ecological measure and should not be interpreted as evidence of improved or worsened dysbiosis by itself. At the individual phylum level, *Actinomycetota*, *Deferribacterota*, and *Pseudomonadota* also showed numerically lower relative abundances following oral administration of *E. limosum*, although none of these differences reached statistical significance (Supplementary Figure S7). Alterations in these phyla have previously been associated with CRC-related dysbiosis and intestinal inflammatory states. In particular, expansion of *Pseudomonadota* has frequently been associated with intestinal inflammation and dysbiosis (Shin et al., 2015), whereas changes in *Deferribacterota*, including members such as *Mucispirillum*, have been reported in experimental models of intestinal inflammation (Berry et al., 2012; Selvanantham et al., 2016). *Actinomycetota* has also been reported to exhibit altered abundance in CRC-associated microbial communities, although this phylum contains functionally diverse taxa and its biological significance is likely context dependent (Gao et al., 2017; Binda et al., 2018). Taken together, the coordinated numerical decreases observed here may suggest that *E. limosum* administration was associated with a broader shift in the intestinal microbial environment rather than a direct selective effect on any single phylum. However, because these differences were not statistically significant and the group size was limited, this interpretation remains exploratory and should be validated in larger cohorts. PICRUSt2 analysis suggested higher predicted abundances of selected butyrate-related orthologs. Because these functions were inferred from 16S rRNA gene profiles, they represent predicted metabolic potential rather than directly measured gene abundance, gene expression, or butyrate production. Differences between the microbiome responses observed here and those reported for El1405 may reflect variation in strain properties, dosing schedules, sample type, collection time, host sex, tumor burden, or analytical pipelines. These observations emphasize the importance of strain-level validation and indicate that tumor suppression by KGMB01548 did not require broad restructuring of overall microbial diversity under the conditions tested.

Several considerations should be noted when interpreting these findings. Although multiple physicochemical and functional results support butyrate as a major candidate contributor to ElCFS activity, its necessity and relative contribution remain unresolved. Selective butyrate depletion followed by add-back, with appropriate controls for accompanying changes in supernatant composition, and broader metabolite profiling are needed to address these questions. In addition, histone H3 acetylation was used as an indicator consistent with HDAC inhibition, and purified butyrate was not evaluated using the same peritumoral administration protocol as ElCFS. The small group size (*n* = 5 per group) limits the precision of the *in vivo* estimates, particularly for the immune and microbiome endpoints. Finally, the immune and microbiome findings should be regarded as exploratory because the T-cell phenotypes were not functionally characterized and the predicted microbial functions were inferred from 16S rRNA gene profiles.

In conclusion, this study provides complementary strain-level and mechanistic evidence for the anti-CRC activity of *E. limosum*. Whereas the previous El1405 study established a broad multi-metabolite, microbiome, and tumor-microenvironment framework, the present work links an independently isolated strain to butyrate-associated cellular responses and antitumor activity, without establishing that butyrate mediates the *in vivo* effects. The convergence of quantitative SCFA analysis, pH-dependent activity redistribution, approximately concentration-matched NaB responses, histone H3 acetylation, glucose-sensitive antiproliferative activity, apoptosis, and genomic capacity for butyrate biosynthesis supports butyrate as a major candidate contributor to ElCFS activity. The local tumor suppression observed after peritumoral ElCFS administration further indicates that secreted metabolites from KGMB01548 retain biological activity outside the oral route. These findings support further investigation of *E. limosum* KGMB01548 and its secreted metabolites as microbiome-derived candidates for CRC prevention or adjunctive intervention.

## Data Availability

The 16S rRNA gene amplicon sequencing data generated from mouse fecal samples have been deposited in the NCBI Sequence Read Archive under BioProject accession PRJNA1528878. The 16S rRNA gene sequence of *Eubacterium limosum* KGMB01548 is available in GenBank under accession number QB001539. The draft whole-genome sequence of *E. limosum* KGMB01548 has been deposited in the GenBank/EMBL/DDBJ databases under accession number JAQSVD000000000.
